# Establishment and characterisation of six human biliary tract cancer cell lines

**DOI:** 10.1038/sj.bjc.6600440

**Published:** 2002-07-02

**Authors:** J-L Ku, K-A Yoon, I-J Kim, W-H Kim, J-Y Jang, K-S Suh, S-W Kim, Y-H Park, J-H Hwang, Y-B Yoon, J-G Park

**Affiliations:** Laboratory of Cell Biology, Korean Cell Line Bank, Cancer Research Center and Cancer Research Institute, Seoul National University College of Medicine, Seoul 110-744, Korea; Department of Pathology, Seoul National University College of Medicine, Seoul 110-744, Korea; Department of Surgery, Seoul National University College of Medicine, Seoul 110-744, Korea; Department of Internal Medicine, Seoul National University College of Medicine, Seoul 110-744, Korea; National Cancer Center, Madu1-dong, Goyang, Gyeonggi 411-764, Korea

**Keywords:** biliary tract cancer, cell line, cell culture, mutation, hypermethylation, E-cadherin

## Abstract

Human cell lines established from biliary tract cancers are rare, and only five have been reported previously. We report the characterisation of six new six biliary tract cancer cell lines (designated SNU-245, SNU-308, SNU-478, SNU-869, SNU-1079 and SNU-1196) established from primary tumour samples of Korean patients. The cell lines were isolated from two extrahepatic bile duct cancers (one adenocarcinoma of common bile duct, one hilar bile duct cancer), two adenocarcinomas of ampulla of Vater, one intrahepatic bile duct cancer (cholangiocarcinoma), and one adenocarcinoma of the gall bladder. The cell phenotypes, including the histopathology of the primary tumours and *in vitro* growth characteristics, were determined. We also performed molecular characterisation, including DNA fingerprinting analysis and abnormalities of *K-ras, p15, p16, p53*, *hMLH1,* *hMSH2*, *DPC4,* β*-catenin, E-cadherin, hOGG1, STK11,* and *TGF-*β*RII* genes by PCR–SSCP and sequencing analysis. In addition, we compared the genetic alterations in tumour cell lines and their corresponding tumour tissues. All lines grew as adherent cells. Population doubling times varied from 48–72 h. The culture success rate was 20% (six out of 30 attempts). All cell lines showed (i) relatively high viability; (ii) absence of mycoplasma or bacteria contamination; and (iii) genetic heterogeneity by DNA fingerprinting analysis. Among the lines, three lines had *p53* mutations; and homozygous deletions in both *p16* and *p15* genes were found three and three lines, respectively; one line had a heterozygous missense mutation in *hMLH1*; *E-cadherin* gene was hypermethylated in two lines. Since the establishment of biliary tract cancer cell lines has been rarely reported in the literature, these newly established and well characterised biliary tract cancer cell lines would be very useful for studying the biology of biliary tract cancers, particularly those related to hypermethylation of *E-cadherin* gene in biliary tract cancer.

*British Journal of Cancer* (2002) **37**, 187–193. doi:10.1038/sj.bjc.6600440 www.bjcancer.com

© 2002 Cancer Research UK

## 

The prognosis of patients with biliary tract cancer is poor despite recent advances in diagnostic and therapeutic techniques (Yamada *et al*, 1995; Yoshida *et al*, 1995). An understanding of the biological nature of this neoplasm is needed to improve the prognosis of these patients. For this purpose, a permanently growing cell line can be the most suitable tool because it is applicable to a variety of experiments, including the understanding of tumour biology (Yano *et al*, 1992; Yamada *et al*, 1995). However, biliary tract cancer cell lines are very rare, a total of 13 cell lines have been reported in the literature. Gene alterations involving in carcinogenesis and cancer progress in the cancers of alimentary tract such as colorectal cancer and pancreatic cancer have been known. However, in biliary tract cancers, few reports have shown genetic changes responsible for oncogene and tumour suppressor gene (Yoshida *et al*, 1995). In the present study, we report the establishment and characterisation of six new human biliary tract cancer cell lines (designated SNU-245, SNU-308, SNU-478, SNU-869, SNU-1079 and SNU-1196). We described cell phenotypes including the *in vivo* and *in vitro* growth characteristics, and DNA profiles for authenticity of each line. We also checked genetic alterations of *K-ras*, *p15*, *p16, p53, hMLH1,* *hMSH2*, *DPC4*, *STK11, E-cadherin, hOGG1, TGF-*β*RII* genes and compared the genetic alterations in tumour cell lines and their corresponding tumour tissues. In these biliary tract cancer cell lines, the methylation status of promoter region in *E-cadherin* gene was also investigated by 5-aza-2′-deoxycytidine treatment and methylation specific-polymerase chain reaction (MS-PCR) after sodium bisulphite treatment.

## MATERIALS AND METHODS

### Cell culture

Cell lines were established from pathologically proven primary biliary tract and ampulla of Vater cancer samples of six Korean patients. Of these, two cancer cell lines originated in extrahepatic bile duct cancer, one in intraheatic bile duct cancer, and one in adenocarcinoma of gall bladder, and two in ampulla of Vater cancer. Solid tumours were finely minced with scissors and disassociated into small aggregates by pipetting. Appropriate amounts of finely minced neoplastic-tissue fragments were seeded into 25 cm^2^ flasks. Tumour cells were initially cultured in ACL-4 medium supplemented with 5% heat-inactivated foetal bovine serum (AR5) (Park *et al*, 1987, 1995). After establishment, cultures were maintained in RPMI 1640 supplemented with 10% heat- inactivated foetal bovine serum. If stromal-cell growth was noted in initial cultures, differential trypsinisation was used to obtain a pure tumour-cell population. Cultures were maintained in humidified incubators at 37°C in an atmosphere of 5% CO_2_ and 95% air. A-431, HeLa, and K-562 cell lines from the American Type Cell Culture (ATCC) and SNU-1 cell line from Korean Cell Line Bank (KCLB) were used for PCR controls. For growth properties and morphology study *in vitro*, population doubling times and cell viability were determined and cells grown on 75-cm^2^ culture flasks were observed daily by phase-contrast microscopy (Park *et al*, 1995).

### Nucleic acid isolation, cDNA synthesis and DNA profiles

Genomic DNA and total RNA were isolated from washed-cell pellets. cDNA was synthesised according to the manufacturer's specifications (Power cDNA synthesis kit; Intron Biotechnology, Seoul, Korea) using 2 μg of total RNA. To compare the genetic alterations between established tumour cell lines and their corresponding tumour tissues, we obtained DNAs from microdissected tumour cells and stromal cells in H&E stained slides of corresponding tumour tissues of each tumour cell line. Approximately 500–1000 dissected tumour or stromal cells were digested using proteinase K method described previously (Hung *et al*, 1995). For DNA-profile analysis, DNA was amplified by PCR at loci containing highly polymorphic microsatellite markers: D1S1586 and D3S1765 (Ku *et al*, 1999).

### Mutation screening in* K-ras, p53, p15, p16, hMLH1* and* hMSH2* genes

Mutation screening of exons 1 and 2 of *K-ras* was performed by DNA sequencing analysis using oligonucleotide primers as previously described (Capon *et al*, 1983). Mutational screening of exons 5 to 8 of *p53* was performed by PCR-based single strand conformation polymorphism (PCR-SSCP) analysis (Kang *et al*, 1996). Primers for PCR-SSCP for exons 1 and 2 of the *p15* gene were synthesised (Orlow *et al*, 1995), and PCR-SSCP primers for exons 1 and 2 of *p16* gene were carried out as described previously (Williamson *et al*, 1995). For deletion analysis of the *p15* and *p16* genes, we amplified the exons of each gene without [α-^32^P]-dCTP. To investigate the genetic status of *hMSH2* and *hMLH1* genes in biliary tract cancer cell lines, PCR-SSCP analysis was used to screen mutations (Liu *et al*, 1994; Han *et al*, 1996).

### Mutation screening in β-catenin, DPC4, STK11, TGF-βRII, and hOGG1 genes

PCR-SSCP analysis to screen mutations of the β-*catenin* gene was carried out by designed-primers for amplification of exons 3, 5, and 6, since mutations reported earlier in the β*-catenin* gene are concentrated at these exons (Kitaeva *et al*, 1997; Iwao *et al*, 1998). To investigate the genetic alteration of the *DPC4* gene, we screened 11 exons by PCR-SSCP (Hahn *et al*, 1998). For mutational analysis of *STK11* gene, the PCR primer pairs were used as described previously (Jenne *et al*, 1998). Mutation screening of the full seven exons of *TGF-*β*RII* gene was also performed by PCR-SSCP analysis (Lu *et al*, 1996). For mutational analysis of *hOGG1* gene, PCR primer pairs and conditions were described previously (Kohno *et al*, 1998).

### Genetic alteration and methylation analysis of* E-cadherin* gene

To investigate mutation, all 16 exons were screened by PCR-SSCP (Berx *et al*, 1996). For determination of the methylation status in *E-cadherin* gene, 5-aza-2′-deoxycytidine treatment and sodium bisulphite modification were employed. For 5-aza-2′-deoxycytidine treatment, cells were seeded at 2×10^5^ cells 75-cm^2^ culture flask on day 0. The cells were treated with 10 μm 5-aza-2′-deoxycytidine for 24 h on days 2 and 5. The medium was changed 24 h after adding 5-aza-2′-deoxycytidine. Cells were harvested on day 8 for analysis of *E-cadherin* expression.

For mRNA expression analysis, cDNA was amplified in a 25 μl PCR reaction using 0.75 μl of the reverse-transcription reaction, the primers and 0.5 units of the Taq DNA polymerase. Both *E-cadherin* and β-*actin* RT–PCR reactions used the same cDNA synthesis. β-*actin* was amplified to control for RNA integrity. The primers for the amplification of *E-cadherin* gene mRNA were described previously (Melki *et al*, 2000). Products were electrophoresed on a 2% agarose gel with ethidium bromide.

The sodium bisulphite reaction was carried out for 16 h at 55°C. Following bisulphite modification, the DNA was ethanol precipitated, dried, and resuspended in 100 μl distilled water. A nested PCR was performed using the nested PCR primers as described previously (Melki *et al*, 2000).

### Cloning and sequencing

Samples showing abnormal bands by SSCP were subjected to DNA sequencing analysis. Fresh PCR products were ligated into PCR™II vectors and subcloned using the TA cloning system (Invitrogen, San Diego, CA, USA). A minimum of 10 individual clones were then pooled and used for DNA isolation. Bi-directional DNA sequencing analysis performed by using dideoxy chain termination method with a T7 DNA polymerase sequencing kit (Pharmacia Biotech Inc., Piscataway, NJ, USA), or directly sequenced using a Taq dideoxy terminator cycle sequencing kit on an ABI 377 DNA sequencer (Perkin-Elmer, Foster City, CA, USA).

## RESULTS

### Culture characteristics

Population doubling times ranged 48 to 72 h, and cell viability ranged from 85 to 94% (Table 1

**Table 1:**
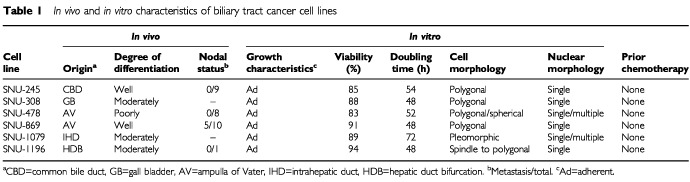
*In vivo* and *in vitro* characteristics of biliary tract cancer cell lines

). All the cell lines were free of contamination with either bacteria or Mycoplasma. The culture success rate was 20% (six out of 30 attempts).

### Morphologic studies

Six carcinoma cell lines derived from biliary tract system were established. The primary tumour of SNU-245 originated from the distal common bile duct. Microscopically, the tumour was composed of well-formed glands lined by a few rows of highly atypical cuboidal cells with lumina containing oeosinophilic material or necrotic cell debris and infiltrated to the stroma. Cell line SNU-308 was established from an adenocarcinoma of a gall bladder. Microscopically, the tumour was composed of well-differentiated neoplastic glands or trabeculae. Two cases of ampulla of Vater carcinoma were obtained from primary tumours. Microscopically, the tumour of SNU-478 was poorly differentiated adenocarcinoma with signet ring cell feature and infiltrated to the pancreas along the interstitial space as a single cell or cell cords. The tumour of SNU-869 was composed of well differentiated adenocarcinoma with focal papillary feature. Five out of 10 periduodenal lymph nodes were involved by this tumour and the tumour infiltrated to the duodenal muscle layer and involved of distal common bile duct. The case of SNU-1079 was obtained from the cholangiocarcinoma arising from right lobe of liver. Microscopically, the tumour of SNU-1079 was composed of irregular nests of tumour cells with focal glandular differentiation, which were separated by fibrous stroma. Cell line SNU-1196 was derived from Klatskin tumour arising in the hepatic duct confluence and invaded to the both intrahepatic duct and common bile duct. Tumour cells had large and vesicular nuclei, and micro or macronucleoli.

**Figure 1:**
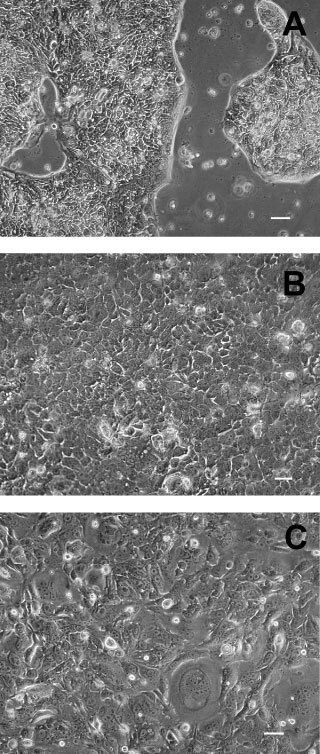
(**A**) Cultured cells of SNU-245 are polygonal shaped adherent cells and arranged in a trabecular pattern with acinar formation. (**B**) Phase-contrast microscopy of SNU-308 cell lines showed polygonal shaped adherent cells with large, round to ovoid, and vesicular nuclei. (**C**) Cultured cells of SNU-478 showed two distinct growth groups : the one growing as a monolayer is polygonal, and the other growing as spherical cells in suspension forms grape-like clusters. Scale bars=50 μM.

In culture, all the cell lines except SNU-478 grew as monolayer of substrate-adherent cells. SNU-245 exhibited trabecular arrangement with acinar formation (Figure 1A

). Tumour cells of SNU-308 was composed of polygonal and relatively uniform cells having large, round to ovoid, vesicular nuclei with prominent nucleoli. Acinar formation was focally seen (Figure 1B). SNU-478 showed two distinct growth pattern, The cells growing as monolayer were polygonal, and cells growing as spherical cells in suspension formed grape-like clusters. The polygonal adherent cells of SNU-478 showed large, round to ovoid, vesicular nuclei with several nucleoli (Figure 1C). SNU-869 composed of polygonal adherent cells grew confluently with frequent acinar formation and floating clumps of tumour cells. SNU-1079 exhibited marked pleomorphic appearance with multiple cytoplasmic processes. Multinucleated bizarre tumour cells were frequently seen. Some cells had numerous cytoplasmic vacuoles. SNU-1196 forming trabecular pattern consisted of spindle to polygonal shaped cells having vesicular nuclei and multiple small nucleoli.

### DNA profiles

**Figure 2:**
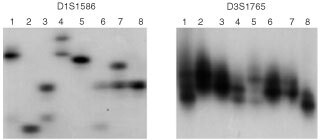
DNA fingerprinting analysis of biliary tract cancer cell lines using highly polymorphic microsatelilte markers. Lane numbers 1 to 8 show cell lines SNU-245, SNU-308, SNU-478, SNU-869, SNU-1079, SNU-1196, HeLa, K-562, and water only. It is evident that each of the six SNU biliary tract cancer cell lines is unique and unrelated.

DNA profiles using two highly polymorphic microsatellite markers (D1S1586 and D3S1765) showed that six biliary tract carcinoma cell lines are unique and unrelated (Figure 2

). These results help exclude the possibility of cross-contamination between the cell lines, as well as by HeLa cell line.

### Mutations in the *p53, p15, p16* and *hMLH1* genes

**Figure 3:**
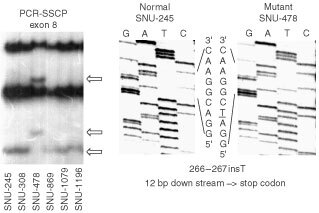
PCR-SSCP and DNA sequencing analysis of exon 8 in the *p53* gene. (left) PCR-SSCP analysis of exon 8. Arrows indicate the shifted bands in SNU-478, as compared with the normal mobility in other cell lines. (right) DNA sequencing analysis of SNU-478.

For *p53* gene, SNU-478 had an adenine insertion at codon 266 of exon 8 resulting in a premature stop codon (TGA) at 12 bp downstream (Figure 3

): SNU-869 harboured a missense mutation from aspartic (GAC) acid to glycine (GGT) at codon 48 of exon 4; SNU-1196 had a missense mutation from asparagine (CGT) to cysteine (TGT) at codon 273 of exon 8. A summary of the results appears in Table 2

**Table 2:**
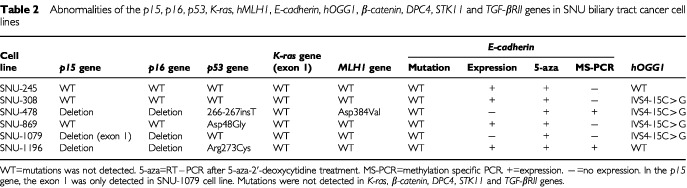
Abnormalities of the *p15*, *p16*, *p53*, *K-ras*, *hMLH1*, *E-cadherin*, *hOGG1*, β-*catenin*, *DPC4*, *STK11* and *TGF-βRII* genes in SNU biliary tract cancer cell lines

.

**Figure 4:**
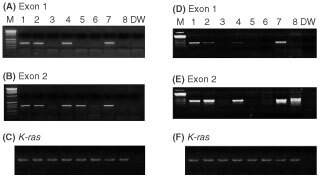
PCR-based deletion analysis of the *p15* and *p16* genes. (**A**,**B**) PCR amplification of the *p15* gene. (**C**) PCR amplification of exon 2 in the *K-ras* gene used as internal control for separate PCR amplification. (**D**,**E**) PCR amplification of the *p16* gene. (**F**) *K-ras* gene for internal control for separate PCR amplification. Lane numbers 1 to 9 show cell lines SNU-245, SNU-308, SNU-478, SNU-869, SNU-1079, SNU-1196, HeLa, K-562, and water only.

There were no band shifts in *p15* and p16 genes by PCR-PCR SSCP analysis. However, there were no PCR amplifications in three lines (SNU-478, SNU-1079, and SNU-1196). By based deletion analysis of each gene, we found that exon 1 of *p15* gene was not amplified in three cell lines (SNU-478, SNU-1079 and SNU-1196) and exon 2 of this gene was not amplified in two cell lines (SNU-478 and SNU-119). We also found that exons 1 and 2 of p16 gene were not amplified in three cell lines (SNU-478, SNU-1079 and SNU-1196) (Figure 4

). The results were listed (Table 2).

In *hMLH1* mutational analysis, only SNU-478 harboured a heterozygous missense mutation from GAT to GTT at codon 384 of exon 12, with resultant amino acid change from aspartic acid to valine (data not shown).

### Mutation analysis in β-*Catenin*, *DPC4*, *hOGG1*, *STK11*, and *TGF-*β*RII* genes

There were no abnormal band shift bands in the β*-catenin, DPC4, STK11*, and *TGF-*β*RII* genes by PCR-SSCP analysis. In *hOGG1* gene, 4 lines (SNU-308, SNU-478, SNU-869 and SNU-1079) were found to harbour abnormal band shift bands in exon 5. By the direct sequencing of DNA fragments corresponding to shifted bands, a C→G nucleotide change at −15 bp from exon 5 was found in all four lines (Table 2).

### mRNA expression and methylation analysis in *E-cadherin* gene

**Figure 5:**
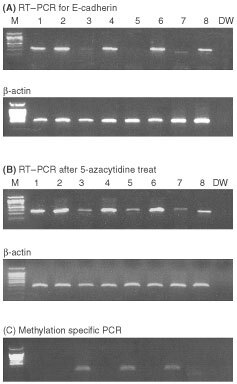
Hypermethylation of *E-cadherin* gene in SNU-biliary tract cancer cell lines. (**A**) RT–PCR analysis of *E-cadherin* gene. Lane numbers (1–9) indicate cell lines: SNU-245, SNU-308, SNU-478, SNU-869, SNU-1079, SNU-1196, SNU-1 (gastric carcinoma cell line, positive control for methylation of *E-cadherin* gene, A-431(negative control), and water only. β-actin is RT–PCR control for mRNA expression. (**B**) RT–PCR analysis after 5-aza-2′-deoxycytidine treatment. It is note that *E-cadherin* gene is re-expressed after 5-aza-2′-deoxycytidine treatment. β-actin is RT–PCR control for mRNA expression. (**C**) Methylation specific PCR analysis after sodium bisulphite modification. It is evident that SNU-478 and SNU-1079 lines are methylated in CpG island of promoter region in the *E-cadherin* gene.

By PCR-SSCP for all 16 exons, abnormal band shifts were not found in all cases. To determine the expression of *E-cadherin* gene in six biliary tract cancer cell lines, we used RT–PCR analysis. SNU-1 and A-431 cell lines were used for negative and positive controls for the expression of *E-cadherin* mRNA. As shown in Figure 5, SNU-245, SNU-308, SNU-869, SNU-1196 and control (A-431) cell lines expressed *E-cadherin* mRNA, whereas SNU-478, SNU-1079 and control (SNU-1) cell lines showed absence of expression (Figure 5A

). Therefore, we determined the methylation status by 5-aza-2′-deoxycytidine treatment and sodium bisulphite modification in these cell lines. After 5-aza-2′-deoxycytidine treatment, *E-cadherin* mRNA was reexpressed in SNU-478, SNU-1079 and control (SNU-1) cell lines (Figure 5B). Methylation specific PCR analysis for CpG island of promoter region in the *E-cadherin* gene after sodium bisulphite modification, DNA fragments were amplified in only SNU-478, SNU-1079 and SNU-1 cell lines (Figure 5C).

### Comparison of genetic alterations in tumour cell lines and their corresponding tumour tissues

Of six biliary tract cancer cell lines, paraffin blocks from four cell lines (SNU-245, SNU-308, SNU-869 and SNU-1079) were available. Tumour cells and stromal cells were dissected, respectively, and DNAs were extracted from these samples. Genetic alterations in DNA of tumour cells were identical to those that had found in tumour cell lines and there were no mutations in constitutional DNAs (data not shown).

## DISCUSSION

Advances in cell culture methods have made it possible to establish a variety of human carcinoma cell lines from surgical and autopsy tissues, peritoneum effusion, and biopsy specimens (Yano *et al*, 1992; Yamada *et al*, 1995). Moreover, because pure cells in cultures can be used for a variety of studies that cannot be carried out using tissue specimens, the study of permanent cell lines established form human cancers has played a major role in our understanding of the biology of cancers. However, cell lines originated from biliary tract cancers have rarely been reported. Ten human extrahepatic bile duct carcinoma cell lines (SK-ChA-1 (Knuth *et al*, 1985), KMBC (Yano *et al*, 1992), and OCUCh-LM1 (Yamada *et al*, 1995), TFK-1 (Saijyo *et al*, 1995), ICBD-1 (Takiyama *et al*, 1998), HBDC (Jiao *et al*, 2000), SCK, JCK, Cho-CK, Choi-CK (Kim *et al*, 2001a)) and three gall bladder carcinoma cell lines (MZ-ChA-1 and Mz-ChA-2 (Knuth *et al*, 1985), OCUG-1 (Yamada *et al*, 1997) have been reported in the world literature.

In this paper, we present six newly established biliary tract cancer cell lines derived from histopathological varied primary carcinomas including a distal common bile duct, gallbladder, two ampulla of Vater, an intrahepatic duct, and a hepatic duct bifurcation tumour. The culture success rate of 20%, reflecting the relative high efficiency of AR5 medium in selective growth of human biliary tract cancers, although the establishment of cell lines from biliary tract cancers, especially cell lines from primary tumours, is very difficult as described above.

Primary tumours revealed morphological heterogeneity including cellular and a growth pattern of original tumours in each tumour type. Cell lines derived from primary biliary tract cancer showed marked heterogeneity in cellular and nuclear morphology, and growth pattern *in vitro*. All six cell lines grew as adherent monolayer, of these, SNU-869 formed prominent domes.

In biliary tract cancers, the role of p53 is still controversial and mutation rate of this gene varied from 33 to 65% according to anatomical site of the biliary tract. In gallbladder carcinomas from Korean, it was reported that 35.7% had mutations in this gene (Kim *et al*, 2001b). In newly established six cell lines, we detect two missense mutations and one frameshift mutation (50%). Two mutations were found to be in exon 8 and one mutation in exon 4.

*p15* and *p16* genes belonging to the cyclin-dependent kinase 4 inhibitory family, have homology and are adjacent to each other. In our six cell lines, simultaneously homozygous deletions of *p15* and *p16* genes, but no point mutation, were found in (SNU-478, SNU-1079 and SNU-1196). In SNU-1079, exon 1 of *p15* gene was deleted and exon 2 was intact. These results indicate that *p15* and *p16* are homozygously deleted preferentially in the region neighbouring the two genes in biliary tract cancer cell lines. Kim *et al (*2001) reported that 30.7% of this gene was mutated in gall bladder carcinomas from Korean. Moreover, the high frequency of deletions of *p15* and *p16* in cell lines indicate the possibility of these genes functioning as tumour suppressor genes in biliary tract cancer as described previously (Yoshida *et al*, 1995).

We also found one heterozygous missense mutation at codon 384 of exon 12 in *hMLH1* gene in one cell line (SNU-478). We screened the microsatellite instability (MSI) status in this line using several MSI markers such as BAT-25, BAT-26 and BAT-40 which were used as a surrogate to indicate MSI status, but failed to find evidence of MSI (Myeroff *et al*, 1995). Although we didn't check this mutation in normal counterpart because of no availability normal tissue, we think that this mutation is polymorphism because this cell line did not show MSI.

A significant number of human carcinomas and cancer cell lines lose sensitivity to negative growth regulation by transforming growth factor β (TGF-β) (Vincent *et al*, 1997). In biliary tract cancers, it was reported that 16% (five out of 32 primary biliary carcinomas) had point mutations in the *DPC4* gene (Hahn *et al*, 1998). In our cell lines, we had not found shifted bands in all samples by PCR-SSCP analysis. Moreover, we found that all cell lines expressed intact *DPC4* mRNA (data not shown). In addition, we did not find genetic alterations in *TGF-*β*RII* gene by PCR-SSCP and RT–PCR analysis, although genetic alterations of the *TGF-*β*RII* gene in biliary tract cancers have been reported in some (Goggins *et al*, 1998; Yazumi *et al*, 2000). We think that our results in six cancer cell lines do not relfect differences in genetics of biliary tract cancers in Korean because the sample size is too small, although we did not found any reports about mutations of *DPC4* and *TGF-*β*RII* genes in biliary tract cancers in Korean.

*E-cadherin* gene on chromosome 16q22.1 encodes a protein product important in the maintenance of the epithelial phenotype mediated by a Ca^2+^-dependent, homotypic cell–cell adhesion. The gene has been termed a ‘metastasis suppressor’ gene, because the E-cadherin protein can suppress tumour cell invasion and metastasis. *E-cadherin* gene expression is reduced or silenced in carcinomas of the breast and liver and many cell lines including those from colon, stomach, and prostate (reviewed by Melki *et al*, 2000). Alterations in DNA methylation patterns are commonly found in essentially all cancers, often with concomitant changes in gene expression. In our study, we have not found mutations in all 16 exons of *E-cadherin* gene by PCR-SSCP. However, we found that *E-cadherin* gene expression was silenced in two cell lines (SNU-478 and SNU-1079) by RT–PCR analysis (Figure 5A). After demethylating agent (5-aza-2′-deoxycytidine) treatment, *E-cadherin* was successfully reexpressed in these two cell lines (Figure 5B). Hypermethylation of *E-cadherin* gene in two cell lines have also been confirmed by methylation specific PCR (MS-PCR) analysis (Figure 5C). These results would be the first report about hypermethylation of *E-cadherin* gene in biliary tract cancers.

Since the establishment of biliary tract cancer cell lines has been rarely reported in the literature, these well-characterised biliary tract cancer cell lines will be useful tools for investigating the biological characteristics of biliary tract cancer, especially those related to the hypermethylation of *E-cadherin* gene in biliary tract cancers.
